# The amount of available food affects diurnal locomotor activity in migratory songbirds during stopover

**DOI:** 10.1038/s41598-019-55404-3

**Published:** 2019-12-13

**Authors:** Andrea Ferretti, Ivan Maggini, Sara Lupi, Massimiliano Cardinale, Leonida Fusani

**Affiliations:** 1 Department of Cognitive Biology, University of Vienna, Althanstrasse 14, 1090 Vienna, Austria; 2 Konrad-Lorenz Institute of Ethology, University of Veterinary Medicine, Vienna, Savoyenstrasse 1, 1160 Vienna, Austria; 3 Department of Aquatic Resources, Marine Research Institute, Swedish University of Agricultural Sciences, Turistgatan 5, 45330 Lysekil, Sweden

**Keywords:** Animal migration, Ecophysiology

## Abstract

Migratory passerine birds fly long distances twice a year alternating nocturnal flights with stopovers to rest and replenish energy stores. The duration of each stopover depends on several factors including internal clocks, meteorological conditions, and environmental factors such as availability of food. Foraging entails energetic costs, and if birds need to refuel efficiently, they should modulate their activity in relation to food availability. We investigated how food availability influences locomotor activity in migrating birds of six passerine species at a spring stopover site in the central Mediterranean Sea. We selected birds with low fat scores which we expected to be strongly motivated to refuel. We simulated stopover sites of different quality by providing temporarily caged birds with different amounts of food to simulate scarce to abundant food. We analysed the diurnal locomotory activity as a proxy for food searching effort. Low food availability resulted in an increased diurnal locomotor activity in almost all species, while all birds showed low intensity of nocturnal migratory restlessness. In conclusion, our study shows that food availability in an important determinant of behaviour of migratory birds at stopover sites.

## Introduction

During spring, millions of migratory birds cover thousands of kilometres from their wintering grounds at equatorial latitudes to their temperate breeding areas^1–3^. Because of the high energy costs of such a long journey and of major ecological barriers along their route, birds break up their migration into several legs, with stopovers in areas where they can sleep^4^ and rebuild their energy stores^5–11^. The duration of the stopovers can amount to 80% of the overall migration time and thus shapes directly overall migration speed and arrival date^12^. As the chances of securing a good breeding territory are increased by arriving early to the breeding grounds^13^, stopover duration has important consequences on reproductive success^12,14–16^. The time spent at each stopover site is influenced by internal factors including endogenous programs and physiological condition^17–23^, and by environmental factors such as meteorological conditions, predation risk, and food availability^24–30^.

Food availability can be a major constraint for restoring the energy stores required for migration – a process called ‘refueling’^6,10,11^. Previous studies have repeatedly shown effects of food availability on nocturnal restlessness − also called Zugunruhe, a good proxy for migratory disposition^19,20,31,32^, and in a few cases on diurnal activity as well^21,31^. For example, in Garden Warblers (*Sylvia borin*) high food intake is associated with low diurnal locomotor activity^21^. Another study on captive Garden Warblers showed a drastic increase in intensity of day time activity during food deprivation, suggesting that day time activity is mostly related to foraging^33^. Similar results have been reported in studies of physiological and behavioural changes in non- migrating songbirds after food restriction^34–36^. Hyperactivity in response to food restriction was ascribed to food-searching behaviour^34,37,38^ and was not linked to levels of stress hormones^35,36^. These findings illustrated the usefulness of the investigation of food intake in caged birds where diurnal locomotor activity functions as a proxy for food searching behaviour.

Most migratory birds forage only during the day and several studies have reported that foraging is in fact the main component of diurnal activity at stopover sites^39–41^. Besides weather factors, the time spent at a stopover site depends on two main factors: condition at arrival^18,28,42–44^ and the speed at which birds can restore their energy reserves, i.e. the fuel deposition rate (FDR)^45^. One of the main factors determining the FDR, besides predation risk that limits foraging activity and intra- and interspecific competition for food, is the extent of food resources^30,46,47^. If predation risk and competition for food were reduced or absent, FRD should basically depend on food availability alone^39,41^. However, there might be a relationship between time and energy invested in searching for food and actual energy gain resulting from foraging. Cohen *et al*. reported such a relationship in a radiotelemetry study on free-living Red-eyed Vireos (*Vireo olivaceus*) which showed a higher diurnal mobility of lean birds in a food-restricted area, suggesting that these birds increased their foraging effort^48^. The study, however, did not quantify or estimate food intake in individual birds.

The aim of our study was to determine how food availability alone influences locomotory activity in controlled conditions where other environmental factors such as predation risk and competition with other birds were removed. In addition, we wanted to control for another main determinant of stopover duration, i.e. condition, by selecting birds with small fat stores. We conducted our study in spring on wild northbound-migrating birds *en route* to their breeding grounds. Birds belonging to six migratory passerine species were caught at a stopover site in the central Mediterranean, a major ecological barrier for Palearctic passerine migrants. Birds were caught with mist nets and transferred into individual cages equipped with a system recording locomotory activity^21,22,31^, and provided with three different amounts of food simulating scarce (1.5 g), intermediate (3.0 g), and abundant (6.0 g) food. We then recorded their locomotor activity for the remaining part of the day and the night and released them the following day. We compared activity between groups of birds that received different amounts of food which simulated different site quality. We hypothesised that the amount of diurnal activity is determined by the extent of food availability, which allows foraging with a higher efficiency. Thus, we predicted that birds offered larger amounts of food will show lower levels of diurnal locomotor activity and yet increase their food intake.

## Results

**Figure 1:**
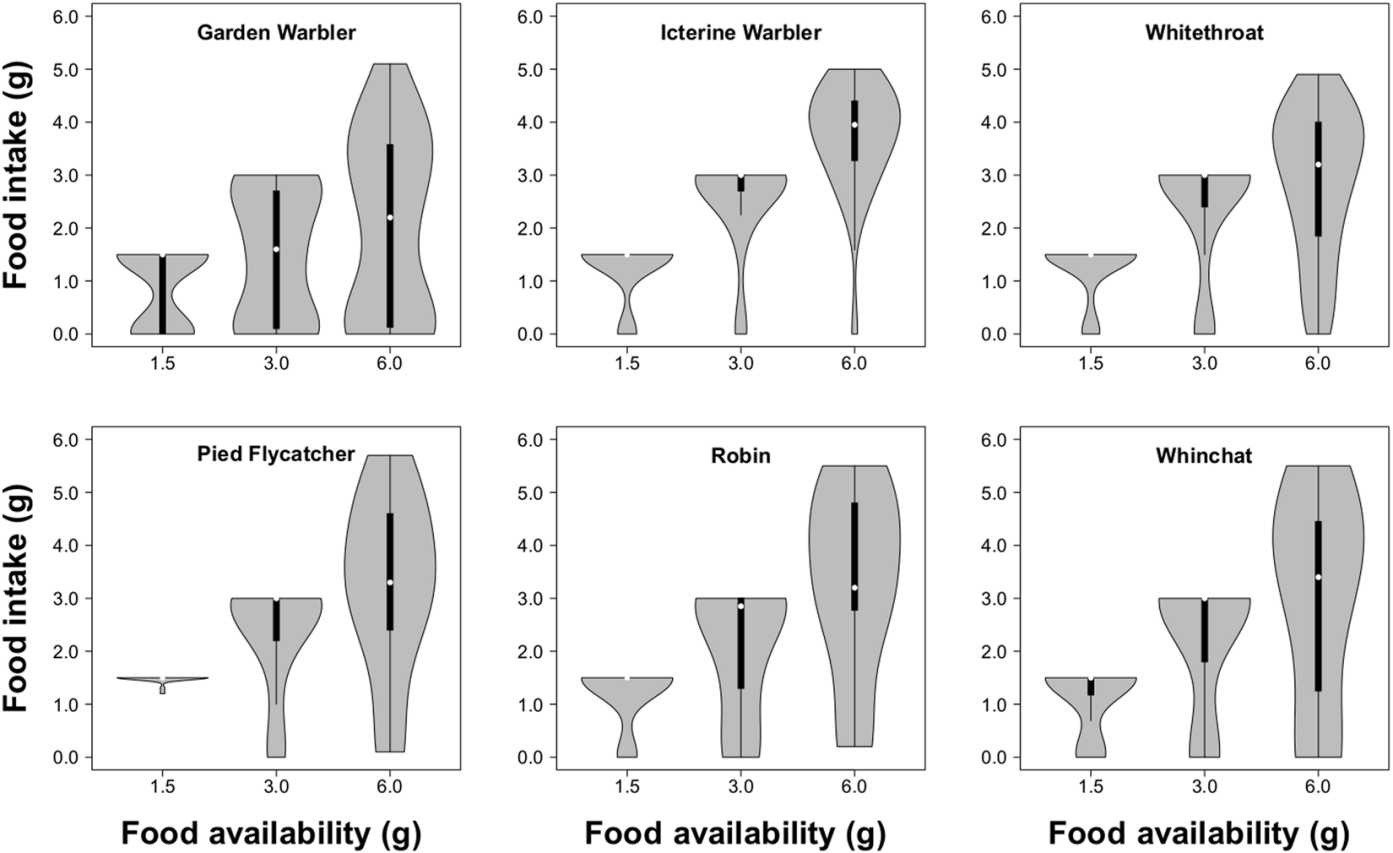
Frequency distribution of food intake in birds provided with different amounts of available food. The violin plots show the median (white dot), interquartile range (black bars) and distribution range (grey area). The shape of the plot indicates the distribution of samples within the range.

In all species, condition was homogenous across food treatments (one-way ANOVA, Garden Warbler: *F*_*2*,*176*_ = 0.175, *p* = 0.840; Icterine Warbler: *F*_*2*,*86*_ = 0.183, *p* = 0.833; Whitethroat: *F*_*2*,*123*_ = 0.085, *p* = 0.918; Pied Flycatcher: *F*_*2*,*69*_ = 0.279, *p* = 0.757; Robin: *F*_*2*,*55*_ = 0.831, *p* = 0.467; Whinchat *F*_*2*,*48*_ = 0.736, *p* = 0.485; Supplementary Material, Fig. S1). In the groups that received 6.0 g of mealworms, no bird ate all available food, and the majority of birds of all species consumed between 3 and 4 g (Fig. 1). Thus, the 6.0 g group effectively received food *ad libitum*. In the 3.0 g groups, the large majority of animals consumed all the food, with the exception of Garden Warblers for which food intake was more evenly distributed (Fig. 1). Considering the distribution of food intake in the 6.0 g group, it appears that most birds of the 3.0 g group ate a substantial amount of food in relation to their maximum capacity. Finally, in the 1.5 g groups most birds ate almost all the food (Fig. 1) and can be considered as food restricted.

**Figure 2:**
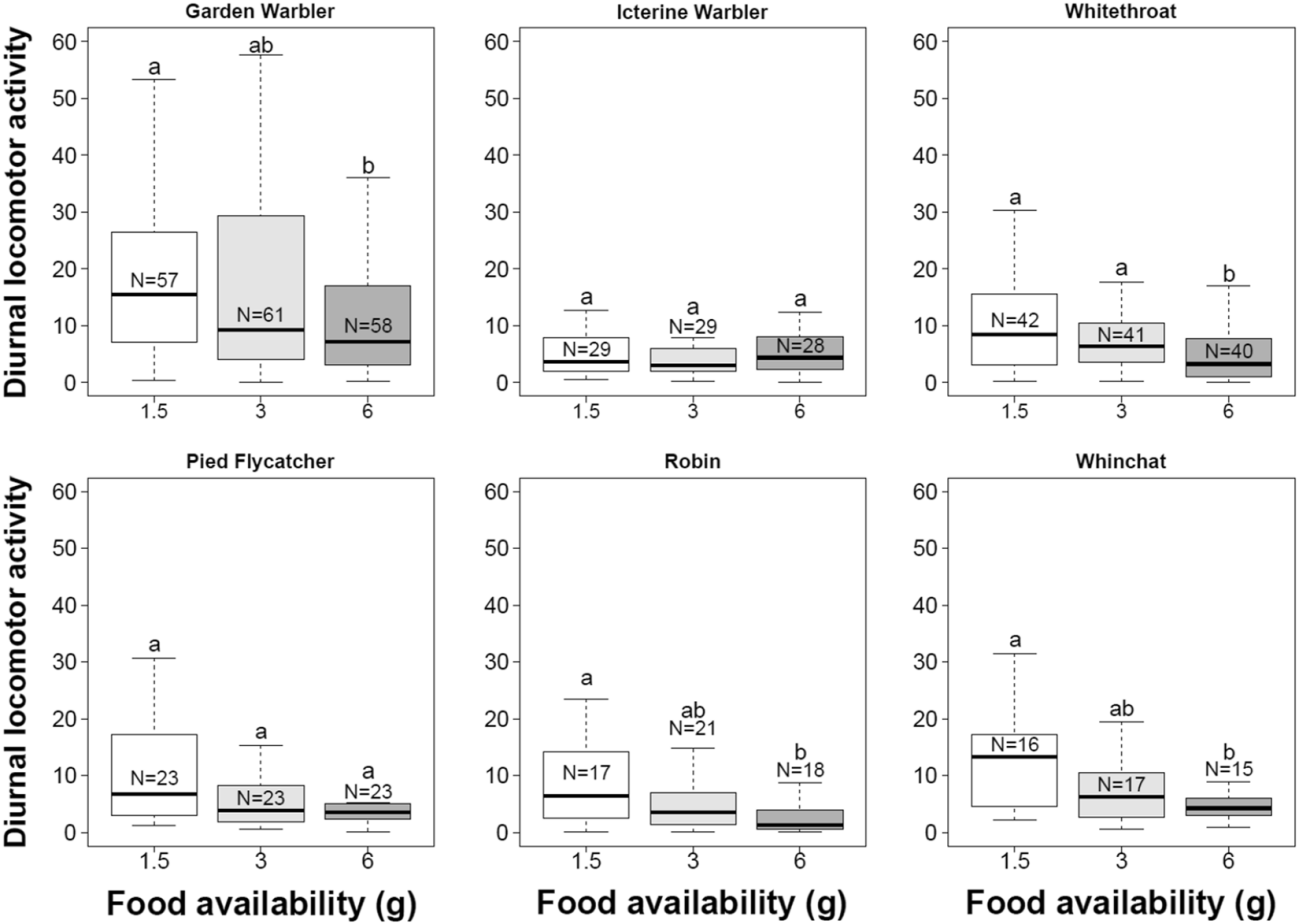
Box plots of diurnal activity (median/interquartiles/range) in birds provided with different amounts of food. Groups labelled with the same letter do not differ significantly from each other (Generalized Linear Models followed by Tukey HSD post-hoc tests; *p* > 0.05). The calculation of the activity is based on the number of activations of an infrared motion sensor and is explained in detail in the methods.

In all our study species, we found a significant effect of the interaction between time of day and food treatment on activity (Supplementary Material, Table S1). The significant interaction likely depended on stronger effects of food treatment during daytime compared to the night hours. When we analysed separately the activity during the different periods of the day (day = diurnal activity; sunset to midnight = Zugunruhe 1; and midnight to sunrise = Zugunruhe 2; see Methods for a detailed description of the variables), we found significant differences in diurnal activity between food treatments in Garden Warblers, Whitethroats, Whinchats, and Robins (Table 1 and Fig. 2). In Pied Flycatchers and Icterine Warblers diurnal activity did not differ among food treatments, although visual inspection of the data suggests that similar levels of activity at the beginning and at the end of the diurnal activity period mask the difference observed in the central afternoon hours (Supplementary Material, Fig. S2). In the species for which we found an effect of food treatment, diurnal activity was significantly lower in the 6.0 g group than in the 1.5 g group (Pairwise post-hoc comparisons: Garden Warbler *p* = 0.020; Whitethroat; *p* < 0.001; Pied Flycatcher *p* = 0.757; Robin *p* = 0.007; Whinchat *p* < 0.001; Fig. 2). In addition, in Whitethroats diurnal activity in the 6.0 g group was also lower than in the 3.0 g group (Pairwise post-hoc comparisons: *p* = 0.004; Fig. 2), and in Whinchats diurnal activity in the 3.0 g group was lower than the 1.5 g group (Pairwise post-hoc comparisons: *p* = 0.016; Fig. 2). We found a significant positive effect of the trap time on diurnal activity in Whitethroats and Whinchats. In Whitethroats, the effect of trap time on diurnal activity was due to a single bird caught at 6:00 which showed much lower activity than the other conspecifics (Supplementary Material, Fig. S3). When we removed this bird from the analysis, the effect disappeared. In Whinchats, the effect of trap time seems to be mainly driven by individuals caught between 6:00 and 7:00 (N = 7; Supplementary Material, Fig. S3). Differences in locomotor activity between these individuals and those caught later in the morning can be due to their history prior to capture – i.e. Whinchats caught in the early morning are less likely to be new arrivals – or to the fact these birds had more time to explore the cage before food was provided.

**Table 1:** Outcome of Generalized Linear Models testing the effects of Food amount, Trap time, Julian Date, Age and Sex on locomotor activity. Statistically significant effects are outlined in bold typeface. Likelihood-Ratio Chi-squared is indicated as LR Chisq.

Species	Variables	DF	Diurnal activity*		Zugunruhe 1*		Zugunruhe 2*	
			LR Chisq	P-value	LR Chisq	P-value	LR Chisq	P-value
Garden Warbler	Food Amount	2	7.200	**0**.**027**	1.457	0.483	0.439	0.803
	Trap Time	1	2.098	0.147	0.945	0.331	0.054	0.817
	Julian Date	1	0.830	0.362	0.005	0.943	3.668	0.055
Icterine Warbler	Food Amount	2	1.292	0.524	6.241	**0**.**044**	0.789	0.674
	Trap Time	1	0.560	0.454	1.346	0.246	0.088	0.766
	Julian Date	1	1.759	0.185	0.095	0.758	0.200	0.655
Robin	Food Amount	2	9.067	**0**.**011**	0.973	0.615	0.022	0.989
	Trap Time	1	2.060	0.151	0.143	0.706	0.010	0.922
	Julian Date	1	0.364	0.546	0.014	0.906	0.038	0.846
	Age	1	0.015	0.904	0.202	0.653	2.485	0.115
Whitethroat	Food Amount	2	18.953	<**0**.**001**	2.381	0.304	1.869	0.393
	Trap Time	1	6.152	**0**.**013**	0.100	0.751	0.147	0.683
	Julian Date	1	1.472	0.225	2.977	0.084	0.504	0.478
	Age	1	1.448	0.229	0.344	0.557	0.028	0.868
	Sex	1	0.436	0.509	0.113	0.737	1.499	0.221
Pied Flycatcher	Food Amount	2	5.209	0.074	0.682	0.711	0.318	0.853
	Trap Time	1	0.797	0.372	0.201	0.654	0.862	0.353
	Julian Date	1	3.430	0.064	5.337	**0**.**021**	4.714	0.299
	Age	1	0.098	0.755	4.187	**0**.**041**	3.726	0.054
	Sex	1	0.003	0.953	0.019	0.890	0.981	0.322
Whinchat	Food Amount	2	14.494	**0**.**001**	1.525	0.467	1.804	0.406
	Trap Time	1	10.047	**0**.**002**	2.428	0.119	0.106	0.745
	Julian Date	1	1.301	0.254	0.070	0.791	0.010	0.919
	Age	1	3.732	0.053	0.036	0.849	0.059	0.809
	Sex	1	0.234	0.629	1.419	0.234	0.003	0.957

*Model: (Dependent variable + 1)^ (λ) ~ Food Amount + Trap Time + Julian Date + Age + Sex.

**Figure 3:**
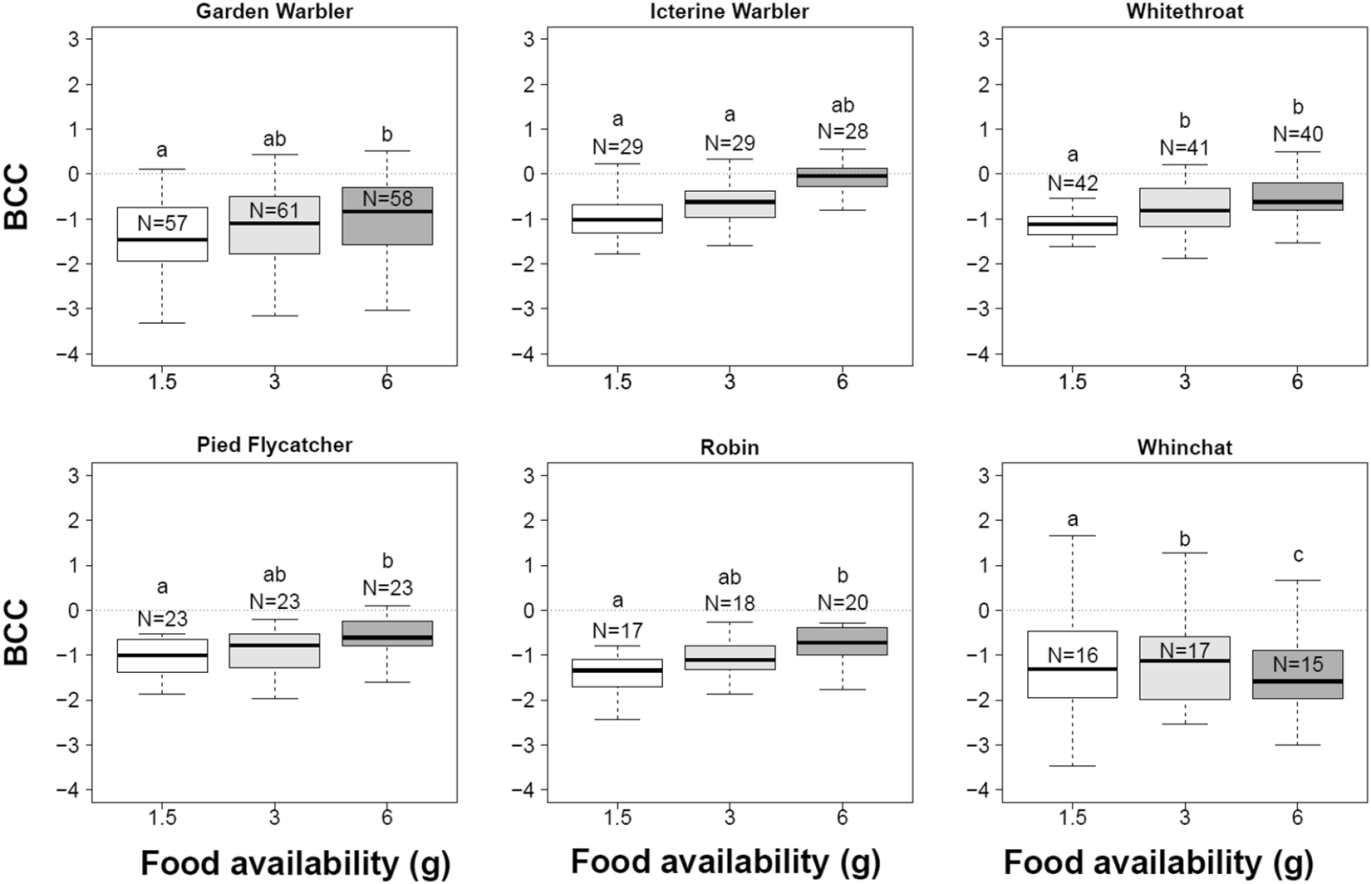
Body Condition Change (BCC; median/interquartiles/range) among species and food availability groups. Groups labelled with the same letter do not differ significantly from each other (Kruskal-Wallis non-parametric ANOVA followed by Kruskal-Dunn post hoc tests; p > 0.05).

None of the species showed intense nocturnal restlessness. Zugunruhe 1 significantly differed among food treatments only in Icterine Warblers, where it was lower in the 1.5 g group compared to the 3.0 g group (Pairwise post-hoc comparisons: *p* = 0.04; Supplementary material, Table S2). No difference in Zugunruhe 1 between food treatments was found in Garden Warblers, Whitethroats, Robins, Whinchats, and Pied Flycatchers. In Pied Flycatchers, we found that Zugunruhe 1 decreased as Julian date and Age increased. No species showed differences in Zugunruhe 2 between food treatments. There was a consistently smaller decrease in condition (Body Condition Change = BCC = condition at release – condition at capture) when a larger amount of food was available (Pairwise post-hoc comparisons between 1.5 g and 6 g: Garden Warbler *p* = 0.013; Icterine Warbler *p* < 0.001; Whitethroat *p* < 0.001; Pied Flycatcher *p* = 0.006; Robin *p* = 0.007, Fig. 3), except for Whinchat (*p* > 0.05). The comparison between species of BCC averaged across treatment groups showed a significantly lower BCC in Icterine Warblers compared to the other species but not Pied Flycatchers and Whitethroats (Pairwise post-hoc comparisons, p-values are summarized in Supplementary material, Table S3, Fig. 3). Finally, although we selected only birds with fat score 1–2, we found differences in condition across species as determined by the Peig method^49^, in that Garden Warblers had significantly higher Peig indexes than all other species while Icterine Warblers showed the lowest indexes (Supplementary material, Fig. S1).

## Discussion

We found that birds receiving a small amount of food were more active during the daytime hours compared to those that received larger amounts. Most of our study species showed significantly lower levels of diurnal activity only when receiving the largest amount of food, i.e. 6.0 g. As no bird in this treatment group consumed all the food, this group can be considered equivalent to an *ad libitum* condition. As expected from birds with low energy reserves, the intensity of nocturnal locomotor activity i.e. Zugunruhe in all experimental subjects was low and we found no effects of food availability on this variable in any of the studied species except for the Icterine Warbler.

These results suggest that the activity of lean migratory songbirds at stopover sites varies depending on the availability of food and supports the hypothesis that lean migrants adapt their stopover behaviour to site quality. In birds with *ad libitum* food, activity was reduced mainly 2–3 hours after food was provided (Supplementary material, Fig. S2). We think that birds that had the largest amount of available food either ate most of the food in the first hours of captivity and thus had a reduced drive to search for food in the following hours, or they learned that food was freely available - either via cognitive or physiological mechanisms - and therefore reduced their search efforts. Our results are in line with previous work on caged passerines which consistently showed an increase of locomotor activity after food restriction^34–36^.

While we are aware of the limitations of studies conducted in captivity with wild birds, we have good reasons to think that our results reveal fundamental patterns of behavioural responses in migrants. First, the main results were similar in all investigated species. Second, our long-term research using the ‘overnight’ approach, which is based on temporarily hosting wild migrants in controlled conditions, has provided in the last decade a number of results which were later confirmed by studies with free-living birds^18,22^. We are well aware that birds might show individual responses to captivity, but relatively large sample sizes, standardized caging conditions, balanced treatment across days, and statistical control of potentially relevant factors (time of day, age and sex when possible) were used to take this aspect into account.

During migration, birds do not always land at stopover sites that guarantee optimal foraging conditions. Landing at a site with suboptimal foraging opportunities motivates them to explore the surrounding areas for food when energy demands are high^50^. This interpretation is consistent with the fact that these movements are often in directions other than the main migratory direction^51^. Some studies suggest that birds actively select their stopover site^52–54^. Species-specific patterns of distribution are consistent in similar habitats across geographical regions^55^, and use of habitat is not always proportional to its availability^56^. There are many extrinsic factors that influence food intake besides its availability, either directly i.e. competition^42^ or indirectly i.e. predation risk^57^. Moreover, there are energetic costs related to food-searching efforts^12,58^. In this scenario, birds that find abundant food should reduce their activity to improve refuelling and reduce predation risk. A radiotelemetry study showed that tagged Red-eyed Vireos explored the site immediately after release and moved further if food availability was low at the release site: migrants moved to patches within the same habitat with more abundant food or to different habitat types that could guarantee higher food availability^48^. These findings are in line with our hypothesis that the effects of food availability on stopover behaviour are related to food searching. Our experimental design does not allow to conclude that the reduced locomotor activity is functional to increase energy saving, although this interpretation appears plausible. For example, reduction of movement as an energy saving strategy has been suggested for migratory birds stopping over in proximity of an ecological barrier^59,60^.

Some of the results of the current study show discrepancies with previous work. For example, we found no effects of food availability on Zugunruhe (except for Icterine Warblers). Previously, we found that Garden Warblers increase Zugunruhe when consuming large amounts of food^21^ and, more generally, when provided with *ad libitum* food^31^. This discrepancy is likely due to differences in experimental design between these studies. The present study focused on birds with low energy reserves which appeared to have a limited capacity to ingest food: within the groups receiving the largest amount of food, i.e. 6.0 g, no bird ate all the worms, and most birds ate around 3.0–4.0 g (Fig. 1). Garden Warblers in migratory conditions eat on average 10.9 ± 0.8 g when refuelling^61^. The lower food intake of birds in the present experiment could be explained by a reduction of the digestive trait, which has been observed in migratory birds after endurance flights^62,63^ and/or by the need to restore proteins first. According to the nutrient-limitation hypothesis, birds use protein reserves during migration and lipid stores can be replenished only after protein^64^. In fact, theoretical models suggest that new arrivals at a stopover site might have low energy stores and low initial rates of energy accumulation because of foraging and settling costs^12,14,30^. The distribution of food intake in this study confirms data collected in previous studies conducted under the same conditions: Robins and Garden Warblers provided with 8 g of mealworms ate more than 6 g only in rare cases^21^. An additional aspect that differs between studies is the type of food used. In the present study, birds were provided with mealworms only, to simulate natural refuelling opportunities at the study site which consist mostly of insects, whereas previous studies used a mixture of dry insect food, banana, and boiled egg^65^. This mixture has a higher content of lipids and carbohydrates (19.0% and 7.7%, respectively) compared to mealworms (8.2% and <1%;^66^). The mixture might thus yield a higher fat deposition rate that would lead to an increase in migratory disposition, as seen in the Garden Warblers studied by Lupi, *et al*.^21^.

Our results provide new information about how food abundance may influence diurnal behaviour at stopover sites. A common feature of all birds caught in Ponza is that they experienced the crossing of a large ecological barrier, the Mediterranean Sea, which offers no refuelling options. This implies the need for a replenishment of fuel stores, especially in birds that reached our study site with depleted reserves. With our experimental design, it was not possible to discriminate between the direct effects of food availability and those consequent to actual food consumption, an aspect that deserves further attention. However, our results suggest that birds with low energy reserves invest in searching for refuelling opportunities when food availability is low. The lower the availability of food, the more these birds will need to allocate time and energy into searching for food. Our results may also help to optimize conservation efforts. Conservation measures are aimed at the protection of valuable stopover habitat^67^. For birds in poor condition, the value of a stopover site is likely to be related to the amount of food that it offers to migrants^48^. A too low profitability could force birds to relocate to different areas^46,51^. Therefore, suitable, food-rich stopover sites should be well-spaced along the migratory route to give migratory birds options to refuel at any stage of their migration, in particular before or after the crossing of major ecological barriers.

### Study site and methods

The study was conducted on Ponza, a small island in the Tyrrhenian Sea, Italy (40°55’ N, 12°58’ E), and is part of a long-term project on stopover biology that our group has started in 2005. Ponza attracts large numbers of African-European migratory landbirds during spring migration. Birds were caught using mist-nets from March to May 2016, and morphological and physiological variables were recorded following standard EURING procedures^68^. One single experienced ringer (MC) scored subcutaneous fat on a 0–8 scale, the size of the pectoral muscle on a 0–3 scale, and measured the length of the third primary feather, a proxy for body size^68^. Body mass was measured to the nearest 0.1 g with an electronic scale and the birds were ringed following EURING guidelines. We used in total 176 Garden Warblers, 86 Icterine Warblers (*Hippolais icterina*), 123 Whitethroats (*Sylvia communis*), 69 Pied Flycatchers (*Ficedula hypoleuca*), 55 European Robins (*Erithacus rubecula*), and 48 Whinchats (*Saxicola rubetra*). Except for the Robin, all these species are long distance migratory passerines that winter in sub-Saharan Africa and breed in Central-Northern Europe. Robins are short and medium distance migrants that winter in Northern Africa and breed in Central-Northern Europe^69^.

We selected birds with fat scores between 1 and 2 which we know to be more motivated to refuel^17,20,31^. Birds with fat score 0 were not used as this class includes individuals in very poor conditions that might not withstand temporary captivity. We estimated condition by using a Scaled mass index where body mass at capture is scaled by the length of the third primary feather^49^. After the ringing procedures, the experimental birds were brought to a recording room where they were placed individually inside custom-made cloth cages (50 × 25 × 30 cm) equipped with perches and were not handled further until release. All birds were hosted in the same room that received natural light through a front glass door, randomizing treatment across the cages to control for possible effects of cage location (i.e. small differences in illumination or temperature). At 12:00 CET we gave the birds 1.5, 3.0, or 6.0 g of live mealworms *Tenebrio molitor* and water *ad libitum*. These amounts of food were chosen based on previous work^17,21,22^ to represent three levels of availability: food is scarce (1.5 g), food is present but not abundant (3.0 g), food is freely available (6.0 g). Birds were randomly assigned to the treatment groups and groups were balanced every day throughout the study period to control for the effects of environmental factors (i.e. temperature, humidity or barometric pressure) on our results. The food trough was removed at sunset and the remaining worms were weighted to calculate food intake. In every cage, an infrared sensor connected to a recording system measured locomotor activity as the total number of movements for each 2-min interval. For analyses, we averaged locomotor activity both for each hour and for three main periods: Day, from 13:00 CET until civil sunset; Zugunruhe 1, from civil sunset until 00:00; and Zugunruhe 2, from 00:00 until civil sunrise. We have traditionally analysed the two halves of the night separately because previous work consistently showed stronger effects of experimental manipulations during the first half^17,20,21,31^. We preferred to use hour or period averages because it allows us to control for day length and to exclude from the analysis data recorded during accidental or planned disturbances, i.e. when a researcher entered the experimental room to remove the food trays. In this case, to be conservative, we excluded also the three sampling periods (3 × 2 min = 6 min) that followed the end of the disturbance. The birds were released on the following morning within one hour from civil sunrise after recording the body mass once again.

### Ethical approval

All experimental procedures including the permission to trap and temporarily hold birds in temporary captivity were authorized by the Regional Government (Determina Regione Lazio N. G02278 of 06 JUN 2015) in accordance with EU and Italian laws, and were communicated to, and performed according to the guidelines of, the Ethic and Animal Protection Committee (ETK) of the University of Veterinary Medicine, Vienna.

### Statistical analysis

First, we tested for the effects of treatment and time of day on locomotor activity using Mixed Effect Models including the individual’s ID as a random factor. Then, we repeated the analysis using the period averages as described above. As the response variables were not normally distributed, we applied a Box-Cox power transformation to meet parametric test requirements. We calculated model-specific λ values for each model which are summarized in Table S4 of the Supplementary Materials. We tested for differences in diurnal activity, Zugunruhe 1 and Zugunruhe 2 between food treatments using Generalized Linear Models. We used this statistical approach because locomotor activity is not a continuous variable but rather a count of the number of activations of the infrared sensor. We controlled for seasonal factors and arrival time by adding Julian date and time of capture as covariates in the model. For species in which sex and age can be determined morphologically, we introduced these two variables into the model. In case of a significant effect of the food treatment, we tested for differences between treatment groups using a Tukey HSD post-hoc test. Moreover, we tested for differences between species in Body Condition Change (BCC = condition at release – condition at capture) as an index of the fuel deposition rate (FDR) using a Kruskal-Wallis one-way ANOVA. In case of significant effects, we performed Kruskal-Dunn post-hoc tests for pairwise comparisons. Statistical analyses were performed with R v.3.5.1^70^ using a significance level of α < 0.05.

## Supplementary information

Supplementary info

## Data Availability

The datasets generated during and/or analysed during the current study are available from the corresponding author on reasonable request.
