# Supplementary material for: The amount of available food affects diurnal locomotor activity in migratory songbirds during stopover
Source: Sci Rep. 2019 Dec 13;9:19027. doi: 10.1038/s41598-019-55404-3 (PMC6910983; doi:10.1038/s41598-019-55404-3)
Supplement: Supplementary file 1 — Supplementary info [file 41598_2019_55404_MOESM1_ESM.pdf]

Supplementary material – Scientific Reports

The amount of available food affects diurnal locomotor activity in migratory songbirds during stopover

Andrea Ferretti[1,2*], Ivan Maggini[2], Sara Lupi[2], Massimiliano Cardinale[3], Leonida Fusani[1,2]

[1] Department of Cognitive Biology, University of Vienna, Althanstrasse 14, 1090 Vienna, Austria

[2] Konrad-Lorenz Institute of Ethology, University of Veterinary Medicine, Vienna, Savoyenstrasse 1, 1160 Vienna, Austria;

[3] Swedish University of Agricultural Sciences, Department of Aquatic Resources, Marine Research Institute, 45330 Lysekil, Sweden

*** Corresponding Author**

Andrea FERRETTI

Department of Cognitive Biology, University of Vienna, Althanstrasse 14, 1090 Vienna, Austria

andrea.ferretti1989@gmail.com

ORCID: 0000-0001-5956-3332

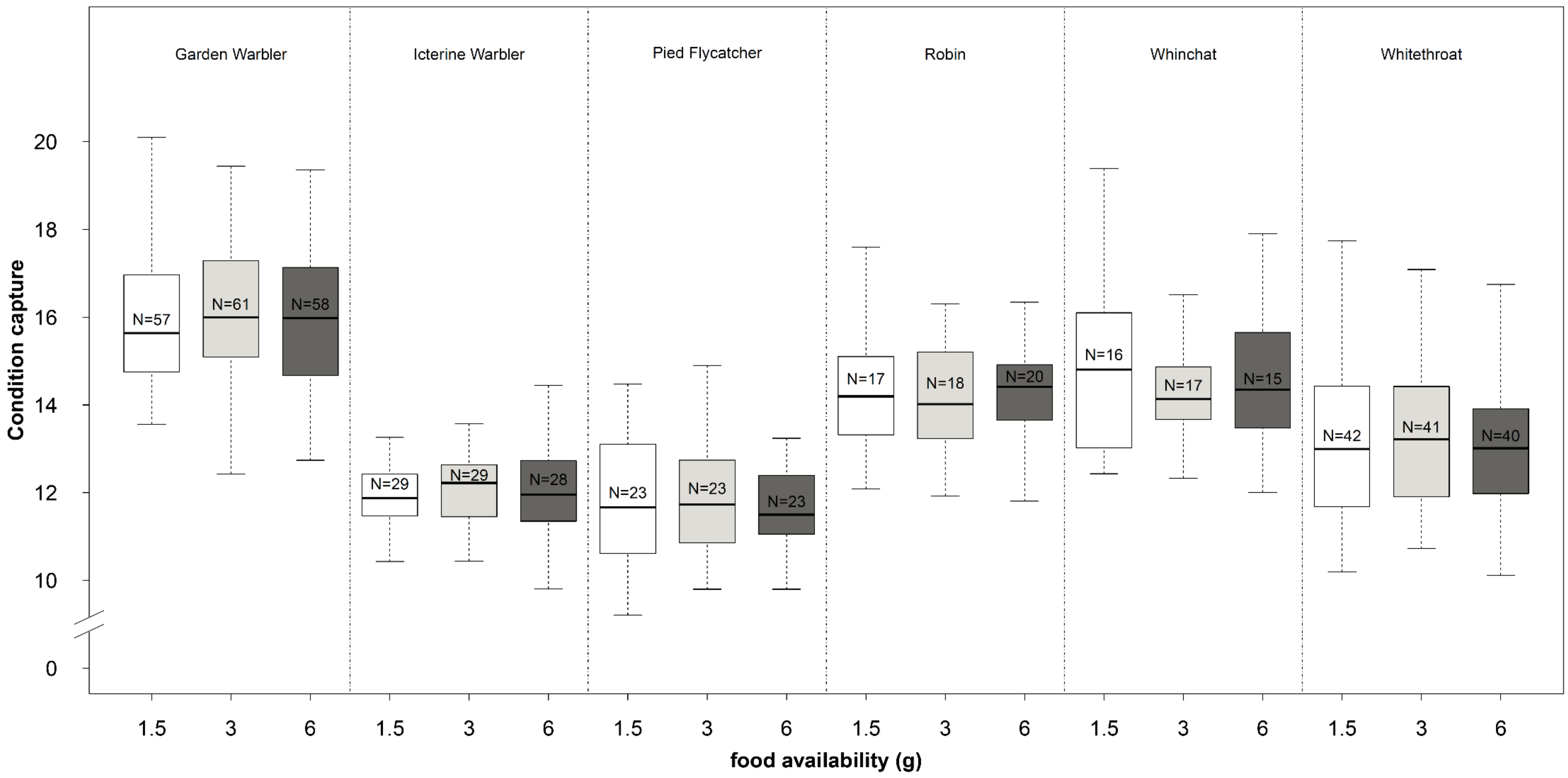

Fig. S1 Body condition at capture by species and amount of provided food. Condition was estimated by using a Scaled mass index where body mass at capture is scaled by the length of the third primary feather[37]. Icterine Warbler and Pied Flycatcher in our sample had lower body condition at arrival than all other species (Kruskal-Wallis non-parametric ANOVA followed by Kruskal-Dunn post hoc tests; $p > 0.05$)

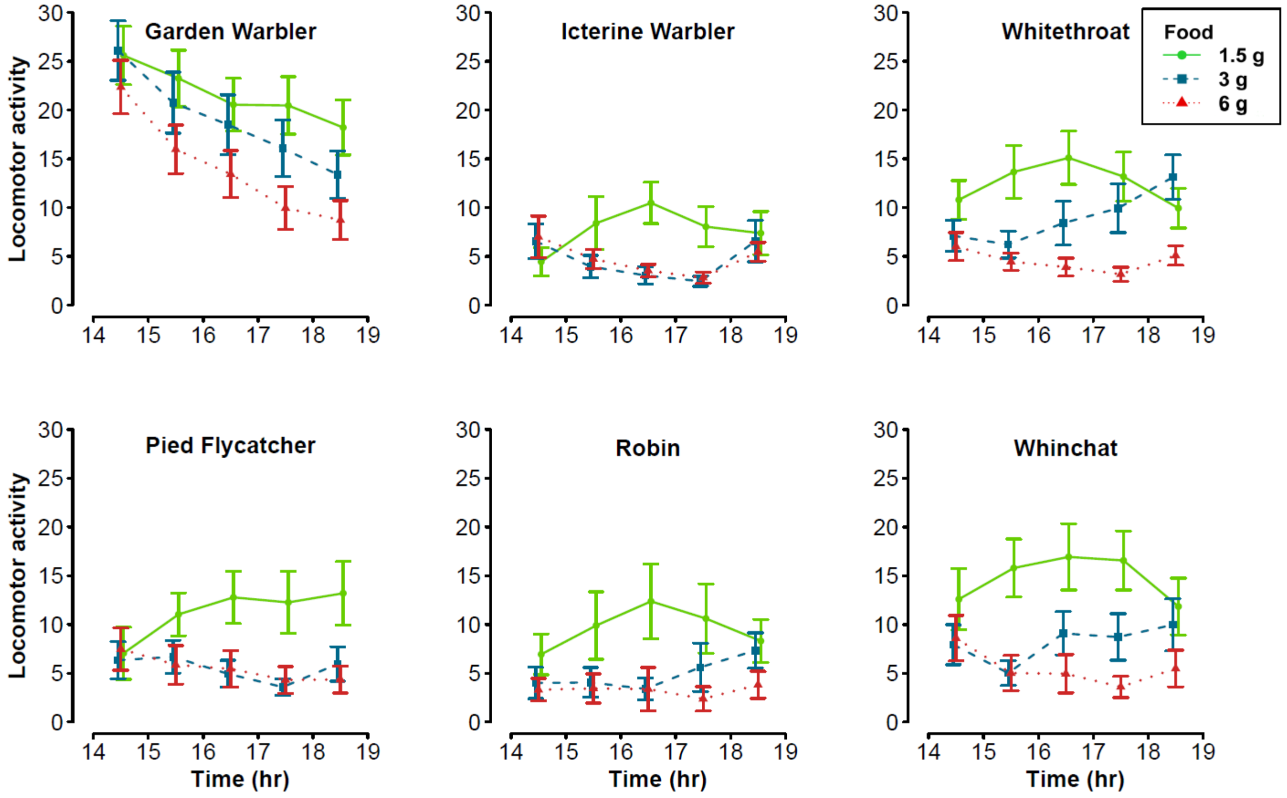

Fig S2 Activity patterns during daylight hours. Each point represents locomotor activity as the average number of movements (± se) per hour. For the graphical representation, hourly intervals were aligned with the time of civil sunset of the first day of experiments.

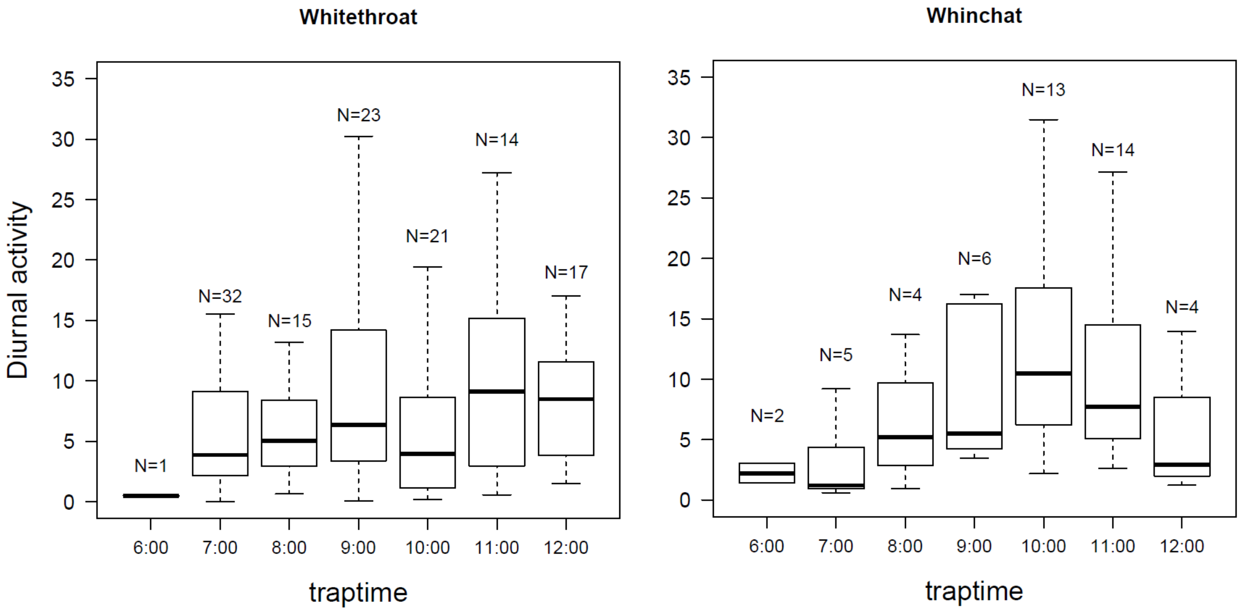

Fig S3 Diurnal locomotor activity and trap time in Whitethroats and Whinchats. In Whitethroats, the effect of trap time on diurnal activity was due to the single bird caught at 6:00. When we removed this bird from the analysis, the effect disappeared. In Whinchats, the effect of trap time seems to be mainly driven by individuals caught between 6:00 and 7:00.

Tab. S1 P-values of Linear Mixed Effect Models considering bird ID as random factor. Significant effects are shown in **bold** typeface.

*Activity ~ Food Amount * time+ (1|RING)*		Chisq	p-value
Garden Warbler	Food amount	3.958	0.138
	Time	625.967	**< 0.001**
	Food amount * Time	46.95	**0.014**
Icterine Warbler	Food amount	0.23	0.891
	Time	81.374	**< 0.001**
	Food amount * Time	70.665	**< 0.001**
Whitethroat	Food amount	11.615	**0.003**
	Time	354.098	**< 0.001**
	Food amount * Time	85.722	**< 0.001**
Pied Flycatcher	Food amount	2.9377	0.230
	Time	354.098	**< 0.001**
	Food amount * Time	85.722	**< 0.001**
Robin	Food amount	6.2869	0.043
	Time	62.0065	**< 0.001**
	Food amount * Time	46.8466	**0.014**
Whinchat	Food amount	5.3813	0.068
	Time	202.862	**< 0.001**
	Food Amount * Time	95.8988	**< 0.001**

Tab. S2 P-values of pairwise post-hoc comparisons for the cases where a significant effect of food treatment on diurnal activity or Zugunruhe 1 was found. Significant differences are shown in bold typeface.

		1.5g vs 3g	1.5g vs 6g	3g vs 6g
		p-value	p-value	p-value
Garden Warbler	Day	0.45	**0.02**	> 0.5
	Zugunruhe 1	NA	NA	NA
	Zugunruhe 2	NA	NA	NA
Whinchat	Day	0.15	**0.02**	> 0.5
	Zugunruhe 1	NA	NA	NA
	Zugunruhe 2	NA	NA	NA
Whitethroat	Day	> 0.5	**0.01**	**0.02**
	Zugunruhe 1	NA	NA	NA
	Zugunruhe 2	NA	NA	NA
Robin	Day	**0.04**	**< 0.01**	> 0.5
	Zugunruhe 1	NA	NA	NA
	Zugunruhe 2	NA	NA	NA
Icterine Warbler	Day	NA	NA	NA
	Zugunruhe 1	**0.04**	> 0.5	0.22
	Zugunruhe 2	NA	NA	NA

Tab. S3 P-values of pairwise post-hoc comparisons of Body Condition Change between species. Statistically significant differences are shown in **bold** typeface.

	Garden Warbler	Icterine Warbler	Pied Flycatcher	Robin	Whinchat
Icterine Warbler	**< 0.001**				
Pied Flycatcher	0.337	0.172			
Robin	1.000	**0.002**	1.000		
Whinchat	1.000	< **0.001**	0.182	1.000	
Whitethroat	**0.047**	0.091	1.000	1.000	0.055

Tab. S4 Lambda (λ) values used for Box-Cox power transformation

		λ
Garden Warbler	Day	0.101
	Zugunrue 1	-0.586
	Zugunrue 2	0.061
Icterine Warbler	Day	-0.141
	Zugunrue 1	-0.545
	Zugunrue 2	-0.505
Whitethroat	Day	0.020
	Zugunrue 1	-0.869
	Zugunrue 2	0.101
Pied Flycatcher	Day	-0.222
	Zugunrue 1	-0.465
	Zugunrue 2	-0.343
Robin	Day	-0.141
	Zugunrue 1	-0.747
	Zugunrue 2	-0.545
Whinchat	Day	0.061
	Zugunrue 1	-0.909
	Zugunrue 2	-0.586
